# Large-scale synthesis of highly emissive and photostable CuInS_2_/ZnS nanocrystals through hybrid flow reactor

**DOI:** 10.1186/1556-276X-9-78

**Published:** 2014-02-17

**Authors:** Jun Lee, Chang-Soo Han

**Affiliations:** 1 School of Mechanical Engineering, Korea University, Seoul, Korea

**Keywords:** CuInS_2_/ZnS nanocrystals, Hybrid flow reactor, Large-scale synthesis, Photostability

## Abstract

We report a high-yield, low-cost synthesis route to colloidal CuInS_2_/ZnS (CIS/ZnS) nanocrystals (NCs) with Cu vacancies in the crystal lattice. Yellow-emitting CIS/ZnS core/shell NCs of high luminescence were facilely synthesized via a stepwise, consecutive hybrid flow reactor approach. It is based on serial combination of a batch-type mixer and a flow-type furnace. In this reactor, the flow rate of the solutions was typically 1 mL/min, 100 times larger than that of conventional microfluidic reactors. This method can produce gram quantities of material with a chemical yield in excess of 90% with minimal solvent waste. This is a noninjection-based approach in 1-dodecanethiol (DDT) with excellent synthetic reproducibility and large-scale capability. The optical features and structure of the obtained CIS/ZnS NCs have been characterized by UV–vis and fluorescence spectroscopies, X-ray diffraction (XRD), X-ray photoelectron spectroscopy (XPS), energy-dispersive X-ray spectroscopy (EDX) and high-resolution transmission electron microscopy (HRTEM). The resulting CIS/ZnS NCs in chloroform exhibit quantum yield (QY) of 61.4% with photoemission peaking at 561 nm and full width at half maximum (FWHM) of 92 nm. The as-synthesized CIS/ZnS NCs were proven to have excellent photostability. The synthesized CIS/ZnS NCs can be a promising fluorescent probe for biological imaging and color converting material for light-emitting diode due to Cd-free constituents.

## Background

Semiconductor nanocrystals (NCs, or quantum dots, QDs) have attracted enormous interests in the last decade as a novel class of material due to their special properties and a wide area of potential applications [1-3], such as biomedical labeling, light-emitting diodes (LEDs), solar cells, lasers, and sensors [4-9]. Among them, cadmium-contained NCs, including CdSe, CdS, and CdTe, have been major research topics due to their good electrical and optical properties, but the inherent toxicity of cadmium limits their applications especially in the biomedical and industrial areas. Commercial products must meet safety standards and comply with regulations. It is partly for this reason, NCs synthesized from the III-V group elements (InP) and from the I-III-VI_2_ group (CuInS_2_ and CuInSe_2_) attracted most interest and lately have reached the performance level comparable to their Cd-based equivalents. These are attractive alternatives to Cd-based QDs due to their nontoxic behavior [10-16].

Among these, CuInS_2_ (CIS) NCs with a bulk band gap of 1.5 eV (827 nm) [17] is selected as an important candidate for optical application and their high potential in solar energy conversion [18-32]. CIS NCs are of particular interest in photovoltaic applications for its high energy conversion efficiency, high absorption coefficient, radiation stability, and low toxicity [15]. Theoretical calculations indicate that the Wannier-Mott bulk exciton radius of CIS NCs is 4.1 nm [23]. It was expected that the CIS NCs with size comparable to Wannier-Mott bulk exciton radius are suitable materials for nanocrystal solar cells and nontoxic luminescent compounds. Thus, it is of very importance to synthesize the CIS NCs with their size smaller than 10 nm. So far, CIS has motivated the development of many synthetic approaches including a solvothermal method [18-21], a precursor decomposition method (thermolysis) [22,23,31], photochemical decomposition [24], and hot injection techniques [25-30,32].

Recently, several works on the synthesis of CIS NCs and an efficient route to highly luminescent CIS/ZnS core-shell NCs were reported [9,33-35]. To improve their photoluminescence (PL) emission properties, shell coating and alloying strategies have been adapted to produce high quality CIS NCs [36-48]. Literature reports have revealed that stoichiometry control is very important in the production of highly luminescent CIS NCs [23,36-48]. In addition, CIS NCs have the characteristic of long PL lifetime, which was thought to be related to intrinsic defects [45]. Based on theoretical models and experimental works on bulk materials, the intrinsic defects of CIS materials have been found to be related to off-stoichiometry effects [46,47]. Although stoichiometry control of CIS NCs has been reported [48], it is still of great interest to further understand off-stoichiometry effects on the physical properties of CIS NCs [49,50].

In the present study, a facile, stepwise hybrid flow reactor method is demonstrated for the first time to synthesize highly fluorescent CIS/ZnS NCs. This reactor is formed based on consecutive combination of the batched mixer and the tube furnace with flowing solution. The flow rate of this reactor was *ca.* 100 times larger than common microfluidics reactors. In order to synthesize high quality CIS/ZnS NCs, we adjusted the concentrations of reactants and the growing temperature. First, CIS core NCs were thermally grown at 210°C for reaction times of 1 min and then ZnS shell overcoating was consecutively conducted via the thermolysis method (320°C for 2 min). The resulting NCs were subsequently overcoated with a ZnS shell using a mixture of zinc acetate, oleic acid, 1-dodecanethiol, and trioctylamine. During the shell growth, the PL quantum yield (QY) strongly increased to values of 20% to 60%. The resulting CIS/ZnS NCs exhibited emissions of yellow with a maximum quantum yield of 61.4%. Both band gap and peak emission energies of CIS/ZnS core/shell NCs were substantially blueshifted compared to that of the original CIS core counterparts (red emission). We provide a simple and reliable synthesis method for CIS-based NCs showing increased fluorescence QY and high photostability.

## Methods

### Materials

Cu(I) iodide (CuI, Aldrich, Yongin City, Kyunggi-do, South Korea, 98%), In acetate (In(OAc)_3_, Aldrich, 99.99%), 1-dodecanethiol (DDT, Aldrich, 98%), 1-octadecene (ODE, Aldrich, 90%), Zn acetate (Zn(OAc)_2_, Aldrich, 99.99%), trioctylamine (TOA, Aldrich, 98%), and oleic acid (OA, Alfa Aesar, 90%) were used without further purification.

### Synthesis of CIS core

In a typical synthetic procedure of CIS NCs with [Cu]/[In] molar ratios of 0.5, CuI (0.0476 g, 0.25 mmol) and In(OAc)_3_ (0.1460 g, 0.5 mmol) were mixed with DDT (5 mL) in a 100 mL two-necked flask, which was followed by the addition of ODE (4 mL). The reaction mixture was degassed under vacuum for 10 min at 150°C. Next, the solution was heated to 210°C for 1 min under nitrogen flow until a deep red colloidal solution was formed. Afterward, the reaction solution was cooled to room temperature.

### Synthesis of ZnS overcoating

To make the Zn and S precursor mixture in flask, 1.46 g of zinc acetate was dissolved in 8 mL of TOA with 4 mL of OA, then mixed with 4 mL of DDT. The resulting mixture was stirred at 100°C for several minutes under vacuum. After cooling down to room temperature, a pump carried the solution (CIS cores plus Zn and S precursors) to furnace (320°C), where ZnS shells were grown on the CIS cores. Finally, the resultant CIS/ZnS core/shell NCs solution was collected at the exit of furnace.

### Purification of CIS/ZnS NCs

The NC dispersions were diluted by the addition of chloroform. The solutions were precipitated with an excess of methanol and 1-butanol. The flocculent precipitate was centrifuged at 4,000 rpm for 2 min and the supernatant decanted. This process was repeated a minimum of three times, and the precipitation was then dried to powder for characterization.

### Characterization

Optical characterization of the CIS/ZnS NCs was carried out using a UV–vis spectrophotometer (Optizen 2120, Mecasys, Korea), a fluorometer (Fluorolog, Horiba Jobin Yvon, France), and an absolute QY measurement system (C-9920-02, Hamamatsu, Japan). X-ray diffraction analysis was performed using a D/MAX Ultima III diffractometer (Rigaku Corporation, Tokyo, Japan) operated at a 40 kV voltage and a 40 mA current with Cu Kα radiation. Data were collected at room temperature in the 5° to 80° range at increments of 0.02°. High-resolution transmission electron microscopy (HRTEM) observations were obtained using a Tecnai G2 F30 S-Twin model (FEI, Hillsboro, OR, USA) at 300 kV. To conduct an investigation by TEM, the NCs were deposited from dilute chloroform solutions onto copper grids with carbon support by slowly evaporating the solvent in air at room temperature. Energy-dispersive X-ray spectroscopy (EDX) analyses of samples were carried out with a Hitachi S-4700 (Hitachi Ltd., Chiyoda-ku, Japan) scanning electron microscopy equipped with an energy-dispersive X-ray analyzer. The photoelectron spectra were obtained with a ESCA-2000 Multilab apparatus (VG Microtech, East Grinstead, West Sussex, UK) using a nonmonochromatic Mg Kα excitation source and a hemispherical analyzer. The residual gas pressure in the chamber during the measurements was approximately 10^−10^ Torr. All spectra were recorded at 90° takeoff angle with a pass energy of 20 eV and at an instrumental resolution of 0.9 eV. The stability of the CIS/ZnS NCs against photooxidation was assessed by placing them into quartz cuvettes with half of the volume being filled with air and continuously irradiating them with a UV lamp (365 nm, distance lamp sample, 10 cm). The photodegradation was monitored by taking PL spectra at certain time intervals.

## Results and discussion

**Figure 1:**
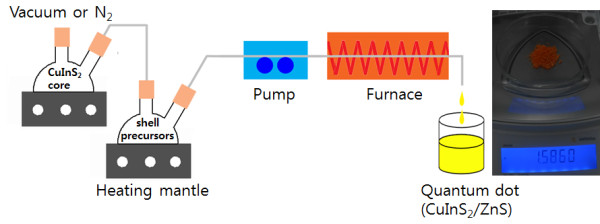
**Schematic diagram showing the hybrid flow reactor.** Optical picture of the sample (*ca.* 1.6 g) obtained from large-scale synthesis.

The scheme of our hybrid flow reactor is shown in Figure 1. The reactor is composed of two flask mixers, one pump, and one furnace. Both flow rate and temperature can be controlled [51]. To produce CIS/ZnS NCs, precursors for the CIS cores are injected into flask mixer. After completing CIS NCs growth, a consecutive ZnS shell overcoating was conducted by adding ZnS stock solution of zinc acetate (8 mmol), OA (4 mL), DDT (4 mL), and TOA (8 mL) into about 10 mL of the CIS NCs crude solution, this time injecting Zn and S shell precursors into furnace (320°C) simultaneously with the CIS core NCs. Using this system, large amounts of CIS/ZnS NCs can be obtained (inset of Figure 1), with colors ranging from yellow to red. Owing to the facileness of our stepwise, consecutive hybrid flow reactor approach, CIS/ZnS NCs are also readily scalable to a larger amount.

**Figure 2:**
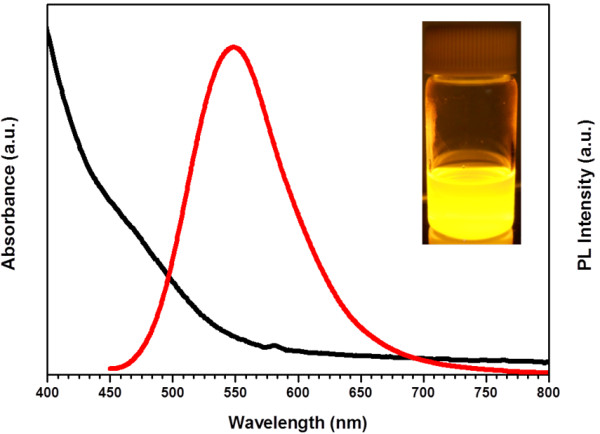
**Absorption and emission spectra of CIS/ZnS NCs.** Photograph of yellow-emitting CIS/ZnS NCs under UV irradiation is shown in the inset in the figure.

As seen from the absorption and PL emission spectra of the CIS/ZnS NCs (Figure 2), the large Stokes shift (i.e., the energy difference between optical band gap and emission peak energy) and broad PL emission are typically observed in chalcopyrite I-III-VI-based semiconductor NCs. The radiative recombination of excited electron–hole pairs in such NCs is associated with deep defect states inside the band gap, being referred to as donor-acceptor pair (DAP) recombination [18,19,52]. These deep traps can be a sulfur vacancy (V_S_), an interstitial copper (Cu_i_), and an indium substituted at a copper site (In_Cu_) as donor states and a copper vacancy (V_Cu_), an indium interstitial (In_i_), and a copper substituted at an indium site (Cu_In_) as acceptor states. Core/shell structured NCs of CIS/ZnS emitted yellow color with a peak wavelength of 561 nm and a broad bandwidth of 92 nm and displayed a good QY of 61.4% owing to the efficient passivation of core surface by a higher band gap of ZnS shell.

**Figure 3:**
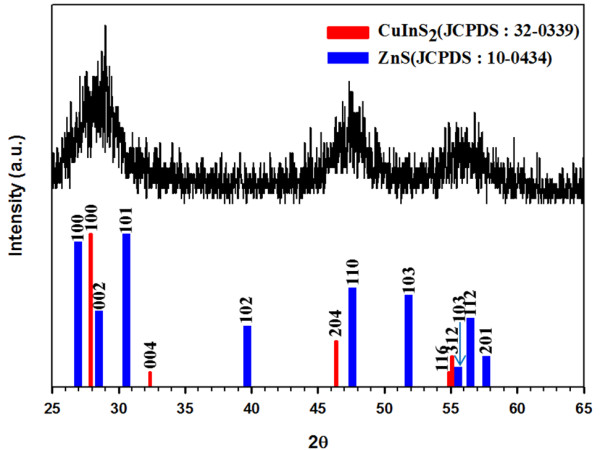
X-ray diffraction patterns of CIS/ZnS NCs.

Figure 3 shows the X-ray diffraction (XRD) patterns of the as-prepared CIS/ZnS core/shell NCs. The powder patterns for CIS (red color) and ZnS (blue color) are also shown for comparison in the bottom to inset. The location of the pattern is in good agreement with the JCPDS reference diagrams in the bottom inset (JCPDS No. 32–0339, CuInS_2_ and 10–0434, ZnS). The diffraction peaks are broadened due to the finite particle size. A strong influence of the ZnS shell on the diffraction is revealed where the XRD pattern is dominated by the NCs. Compared with the standard diffraction data of chalcopyrite CIS, the three major peaks are observed to shift to larger angles, suggesting that the crystal structure of the CIS NC was slightly altered by ZnS shell coating. It is the strain between core and shell that cause this behavior, which caused by the lattice mismatch between CIS and ZnS nanoparticles. Similar studies and analysis were reported for CdSe/ZnS shell structures [53,54].

**Figure 4:**
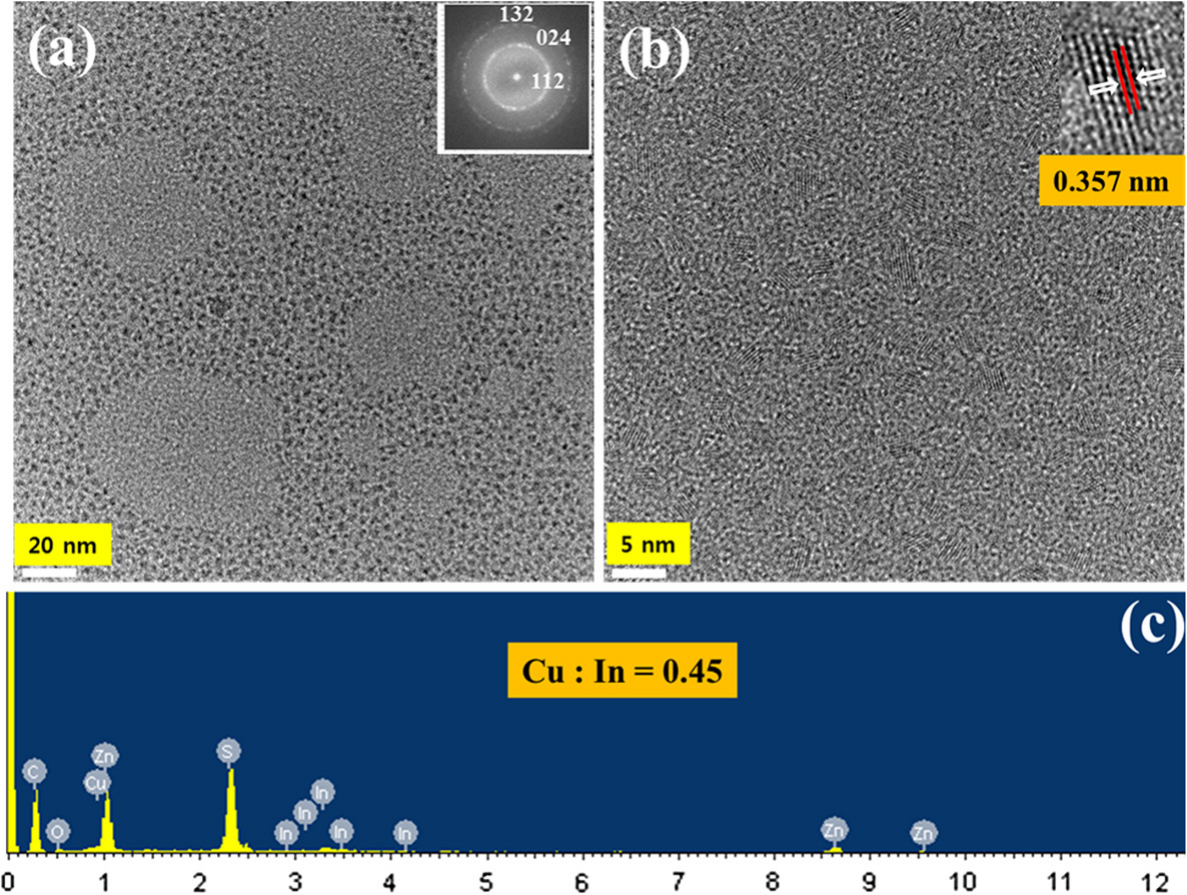
**TEM images and EDX spectrum of CIS/ZnS NCs. (a)** Low-resolution and **(b)** high-resolution TEM images of CIS/ZnS NCs. **(c)** EDX spectrum of the CIS/ZnS NCs.

Images in panels a and b in Figure 4 show representative low-resolution and high-resolution TEM images of CIS/ZnS NCs, respectively. The high-resolution TEM image of the CIS/ZnS NCs in Figure 4b displays clear lattice planes and good crystallinity with diameters of 4 to 5 nm. The TEM analysis shown in the inset of Figure 4a shows the corresponding electron diffraction pattern of indexed showing rings that are consistent with the [112], [024], and [132] reflections for CIS/ZnS NCs. The EDX spectrum in Figure 4c indicates that the sample is composed of copper, indium, sulfur, and zinc. Actual Cu/In composition ratio of CIS/ZnS NCs with Cu/In = 0.5 was calculated to be 0.45 which was almost identical with the solution molar ratio used for CIS/ZnS NCs synthesis.

**Figure 5:**
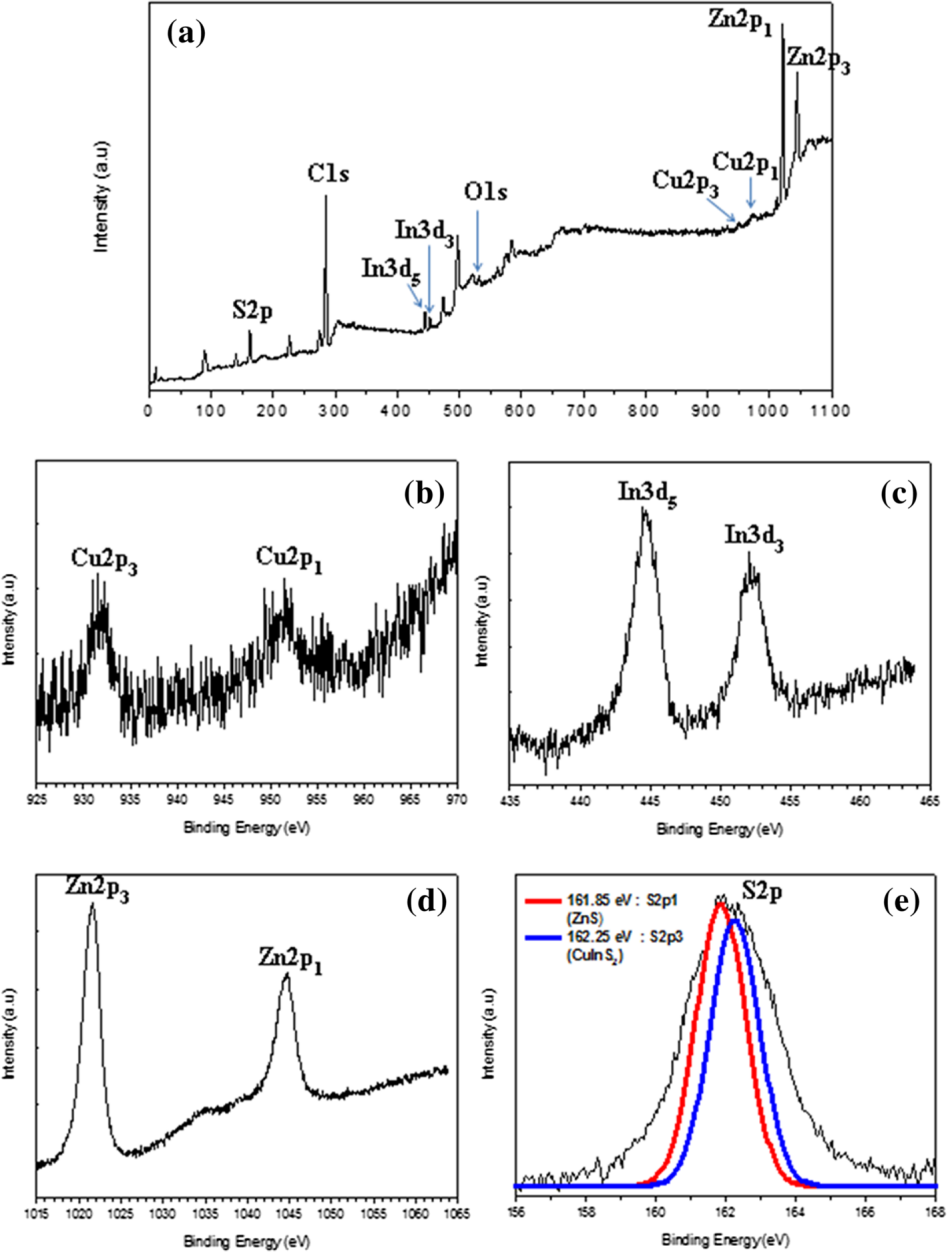
**XPS spectra of the obtained CIS/ZnS NCs. (a)** XPS survey spectrum of CIS/ZnS core/shell NCs. **(b)** The XPS spectrum of Cu 2p. **(c)** The XPS spectrum of In 3d. **(d)** The XPS spectrum of Zn 2p. **(e)** The XPS spectrum of S 2p of the CIS/ZnS NCs.

The compositions and valence states of the obtained CIS/ZnS NCs are also further investigated by the X-ray photoelectron spectroscopy (XPS) as shown in Figure 5. The survey spectrum (Figure 5a) indicates the presence of Cu, In, S, Zn, and C as well. The C is likely due to the capping agents and the contamination as a result of the sample exposure to atmosphere. The Cu 2p, In 3d, and S 2p core levels are examined, respectively. The Cu 2p core splits into 2p3/2 (931.5 eV) and 2p1/2 (952.3 eV) peaks as shown in Figure 5b and is in good accordance with those reported in literature [3,27], suggesting that the copper valence state in the CIS/ZnS NCs is +1. Similarly, In 3d shown in Figure 5c splits two peaks at 444.5 and 452.1 eV, consistent with a valence of +3. The spectrum of the Zn 2p in Figure 5d is divided into 2p3/2 (1,021.7 eV) and 2p1/2 (1,044.8 eV) peaks, confirming the presence of Zn atoms in NCs, and the S 2p in Figure 5e has doublet peaks of S 2p1/2 (161.9 eV) and 2p3/2 (162.3 eV), assigned to a valence of −2. Moreover, the S 2p core level spectrum (Figure 5e) has two peaks at 161.9 eV for ZnS and at 162.3 eV for CIS, which are separated by an energy difference of 0.4 eV that is the same to that for CIS/ZnS [55]. The XPS data of our sample agree well with the previous reports on CIS/ZnS NCs [27,56]. The quantification of those peaks gives the stoichiometric ratio of Cu:In as 0.51:1. Based on the XRD, TEM, and XPS data, it can be concluded that the CIS/ZnS NCs with a size of 4 to 5 nm have been successfully synthesized by a facile flow reactor approach.

**Figure 6:**
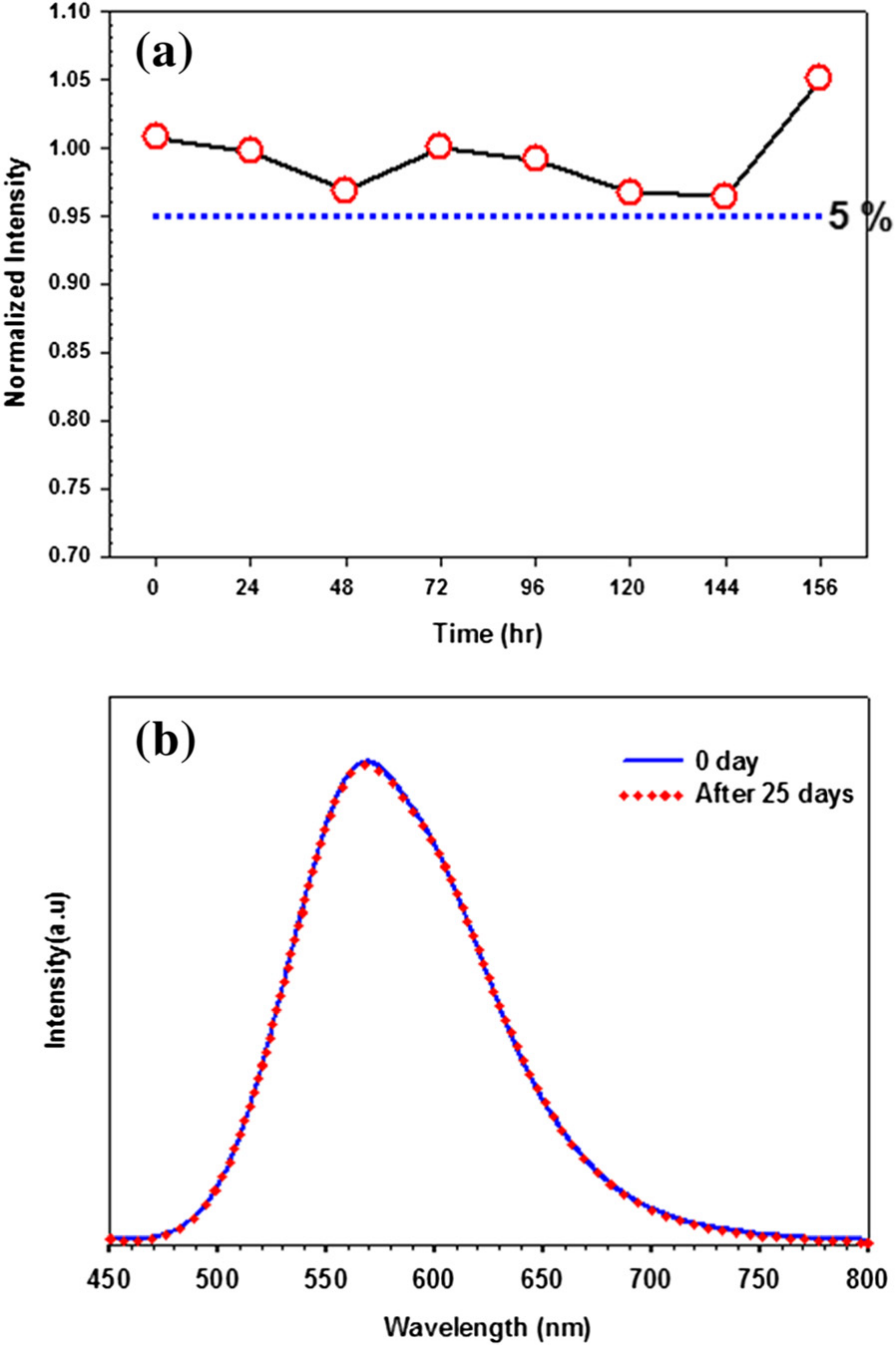
**The PL intensity and emission measurements of CIS/ZnS NCs. (a)** The evolution of PL intensity of CIS/ZnS NCs and **(b)** emission measurements after 25 days in the dark under 365 nm radiation.

From a green chemistry viewpoint, the CIS/ZnS NCs does not contain heavily toxic elements such as cadmium (Cd), mercury (Hg), lead (Pb), and arsenic (As), which is propitious to biomedical imaging, assays, and color converting. Considering the photostability of the CIS/ZnS NCs is critical for their practical applications. We utilized a UV lamp for continuous intensive excitation at 365 nm for 156 h and got the photobleaching graph of the CIS/ZnS NCs (Figure 6a). From Figure 6b, we clearly observed that the fluorescence emission intensity of the CIS/ZnS NCs was almost constant during the 25-day irradiation process. The excellent photostability and nontoxic composition allow CIS/ZnS NCs to be used as a new class of fluorescent labels in biomedical imaging and color converting.

## Conclusions

In summary, highly luminescent CIS/ZnS core/shell NCs with a quantum yield of 61.4% were synthesized on a large scale using a hybrid flow reactor in a simple, one-step process. XRD, XPS, EDX, and HRTEM characterizations show that the fabricated CIS/ZnS NCs are of high quality. This newly developed synthetic methodology is ideal in a number of ways as it can be easily altered to yield a high-quality product on a gram scale with low loss, is highly reproducible, and is based on green chemistry. In the present work, the photostability of high-quality CIS/ZnS NCs was investigated at ambient condition both under UV irradiation and in the darkness. The as-synthesized CIS/ZnS NCs were proven to have excellent photostability. In addition, this method can also be simply extended to AgInS_2_, Zn_*x*_(CuIn)_1-*x*_S_2_ and CuGa_*x*_In_1-*x*_S_2_ systems, which are also of interest for light emitting, biolabel, and solar harvesting applications.

## Competing interest

The authors declare that they have no competing interests.

## Authors’ contributions

JL performed the experiments and analyzed the results. CSH conceived and designed the experiments, analyzed the results, and participated in writing the manuscript. Both authors read and approved the final manuscript.
